# *lac‐1* and *lag‐1* with *ras‐1* affect aging and the biological clock in *Neurospora crassa*

**DOI:** 10.1002/ece3.2554

**Published:** 2016-10-24

**Authors:** John K. Brunson, James Griffith, Daneisha Bowles, Mary E. Case, Jonathan Arnold

**Affiliations:** ^1^ Center for Marine Biotechnology and BiomedicineJ. Craig Venter Institute—West Coast CampusUniversity of California San Diego Scripps Institution of OceanographyLa JollaCAUSA; ^2^ College of Agricultural and Environmental SciencesUniversity of GeorgiaAthensGAUSA; ^3^ Genetics Department University of GeorgiaAthensGAUSA

**Keywords:** aging, balancing selection, biological clock, ceramide, circadian rhythms, *lac‐1*, *lag‐1*, *Neurospora crassa*

## Abstract

Using an automated cell counting technique developed previously (Case et al., *Ecology and Evolution* 2014; 4: 3494), we explore the lifespan effects of *lac‐1*, a ceramide synthase gene paralogous to *lag‐1* in *Neurospora crassa* in conjunction with the *band bd* (*ras‐1*) gene. We find that the replicative lifespan of a *lac‐1* ^KO^ *bd* double mutants is short, about one race tube cycle, and this double mutant lacks a strong ~21‐hr clock cycle as shown by race tube and fluorometer analysis of fluorescent strains including *lac‐1* ^*KO*^. This short replicative lifespan phenotype is contrasted with a very long estimated chronological lifespan for *lac‐1* ^KO^ *bd* double mutants from 247 to 462 days based on our regression analyses on log viability, and for the single mutant *lac‐1* ^KO^, 161 days. Both of these estimated lifespans are much higher than that of previously studied WT and *bd* single mutant strains. In a *lac‐1* rescue and induction experiment, the expression of *lac‐1* ^*+*^ as driven by a quinic acid‐dependent promoter actually decreases the median chronological lifespan of cells down to only 7 days, much lower than the 34‐day median lifespan found in control *bd* conidia also grown on quinic acid media, which we interpret as an effect of balancing selection acting on ceramide levels based on previous findings from the literature. Prior work has shown phytoceramides can act as a signal for apoptosis in stressed *N. crassa* cells. To test this hypothesis of balancing selection on phytoceramide levels, we examine the viability of WT, *lag‐1* ^KO^ *bd*, and *lac‐1* ^KO^ *bd* strains following the dual stresses of heat and glycolysis inhibition, along with phytoceramide treatments of different dosages. We find that the phytoceramide dosage–response curve is altered in the *lag‐1* ^KO^ *bd* mutant, but not in the *lac‐1* ^KO^ *bd* mutant. We conclude that phytoceramide production is responsible for the previously reported longevity effects in the *lag‐1* ^KO^ *bd* mutant, but a different ceramide may be responsible for the longevity effect observed in the *lac‐1* ^KO^ *bd* mutant.

## 1 Introduction

Our previous publication revealed that the *longevity assurance gene* (*lag‐1*), a gene encoding a ceramide synthase, not only is a longevity gene but also exerts control over the biological clock in the model organism *Neurospora crassa* (Case et al., 2014). Circadian rhythms and biological clocks play important roles in diverse cellular processes across the tree of life (Bell‐Pederson et al. 2005). Recently, transcriptomics studies screening a wide variety of marine bacteria have demonstrated widespread diel synchronization of the biota inhabiting the world's oceans (Ottesen et al., 2014). At the organismal level, transcriptomic studies using microarray technologies have shown that nearly twenty‐five percent of the genes in the *N. crassa* have a circadian response, revealing that the biological clock has evolved as an important mechanism for controlling many biochemical processes (Dong et al., 2008). These same transcriptional studies revealed a transient response in transcription levels of *lag‐1* under knockdown of *white collar‐1* (*wc‐1)*, an important *N. crassa* clock gene (Case et al., 2014). These findings spurred our interest in studying the longevity and clock effects of *lag‐1* knockouts, along with our current study on knockouts of the *lag‐1* paralog, *longevity assurance cognate‐1* (*lac‐1*).

Ceramides represent a family of diverse yet closely related signaling sphingolipids with over 200 structural variants found in mammals alone (Hannun & Obeid, 2011). Phytoceramide is one of the two major classes of ceramides found from fungal populations; the other class includes the monohexose‐modified ceramides (Merrill, 2002) (Warnecke & Heinz, 2003). Phytoceramide has been demonstrated to play an important role in mediating and possibly decreasing instances of stress‐induced cell death, but the decrease in stress‐related cell death only occurs at specific dosages, beyond which phytoceramide can have a lethal effect (Plesofsky, Levery, Castle, & Brambl, 2008). This is partial evidence for balancing selection on a quantitative trait, namely the level of phytoceramide synthesis. The evolution of balancing selection on quantitative traits is an extensive subdiscipline unto itself and may provide a useful framework to examine the evolved thresholds of phytoceramide synthesis (Schluter, 1988) (Stinchcombe, Agrawal, Hohenlohe, Arnold, & Blows, 2008) (Lerner, 1954).

Both *lag‐*1 and *lac‐1* have been demonstrated to play important roles in ceramide synthesis in yeast, acting as components of ceramide synthases responsible for converting dihydrosphingosine or phytosphingosine into ceramides (Guillas et al., 2001). From our recent publication, we found that a *lag‐1* knockout combined with a *band* (*bd)* mutation in *N. crassa (more recently identified with the mammalian protooncogene ras‐1,* Belden et al., 2007) seemed to halt the proper functioning of the clock, as revealed by race tube experiments and 48 hour expression profiling of the *frq* clock oscillator (Case et al., 2014). However, this is not the first known instance of a gene implicated in lipid metabolism exerting a unique effect on clock function. For example, mutants in the lipid metabolism genes, *chain elongation* (*cel*) and *choline requirer* (*chol‐1),* have both been shown to create lipid deficiencies and subsequently maintain circadian rhythms in double mutants, such as *chol‐1, wc‐1* lacking a critical clock gene (*e.g*., *wc‐1*)in *N. crassa* (Lakin‐Thomas & Brody, 2000). Subsequent work using a luciferase (*luc)* recorder under the control of *frq* promoter (*frqP*) found 22‐hr periodicity for the luciferase tracings in the *bd*,* chol‐1*,* csp‐1*,* frqP:luc* mutant under choline starvation while the banding pattern displayed a long period characteristic of *chol‐1* (Shi, Larrondo, Loros, & Dunlap, 2007). This raised the question of whether or not the metabolic oscillator tied to *chol‐1* might be independent of the FRQ‐WCC oscillator. With regard to viability, a double knockout in *LAG‐1* and *LAC‐1* has been demonstrated to cause lethality in yeast. However, the same study found that expression of *LAG‐1*, and therefore the rate of sphingolipid and ceramide metabolism, has a nuanced effect on yeast longevity. A moderate increase in *LAG‐1* expression increases longevity, but too much *LAG‐1* transcript has a negative effect on viability (Jiang, Kirchman, Allen, & Jazwinski, 2004). This is further evidence for balancing selection on ceramide synthesis. However, the lipid metabolism hypothesis for the link between aging and the clock is not the only model in existence. Other models have demonstrated a possible role played by reactive oxygen species in both aging and clock function (Gyongyosi and Kaldi 2013) and more recently in the Ras‐Erk‐ETS signaling pathway (Slack et al., 2015). A variety of metabolic linkages may exist between the clock and aging (Judge, Griffith, & Arnold, 2017). The *lag‐1* gene is particularly interesting because it seems to affect directly both aging and the clock, suggesting a possible connection between the two processes.

We are therefore interested to see whether the *lac‐1* gene has similar effects on longevity or the clock to those exerted by *lag‐1*. Aging as defined from an evolutionary perspective is the persistent decline in “fitness components due to internal physiological deterioration” (Rose, 1991). Do conidia age? If they were dormant, one might argue they cannot age. Recent single cell work, however, reveals that conidia are metabolically active and have a working single cell oscillator (Deng et al., 2014, 2016). They are not dormant. The internal physiological deterioration of conidia under stress has been linked to the apoptotic signal of phytoceramides in *N. crassa*, a metabolic product of *lac‐1* (Plesofsky et al., 2008). We will measure a fitness component through viability curves. It is then reasonable to presume that conidia do age, if viability curves of conidia decline much as those do for *S. cerevisiae* cells.

Lifespan can be measured in two ways (Bitterman, Medvedik, & Sinclair, 2003; Case et al., 2014). Replicative lifespan is simply how many serial transfers from one race tube to the next can be carried out. Chronological lifespan is how long a conidium lives in a particular medium. We have been using an automated cell counting method in order to assess conidial chronological lifespan in *N. crassa* mutants as an alternative to the traditional plating method used for over 50 years (Berkes, Chan, Wilkinson, & Paradis, 2012). This automated cell counting method is less laborious and more accurate than previously used methods that involve plating conidial suspensions, counting colonies, and then estimating the total number of viable cells per ml in the solution based on the number of colonies formed on plates (Munkres & Furtek, 1984b). Estimates of chronological lifespan can be extracted from measurements of conidial viability (Table 2) (Munkres & Furtek, 1984a). Based on our comparison of the two methods, we find that the automated cell counting method is five times more precise than the plating method in estimating viability (Case et al., 2014).

Here, we further evaluate the recently developed automated cell counting method by testing the single gene effect of the ceramide synthase encoding gene *lac‐1* using a quinic acid‐inducible promoter. We also use race tubes and an mCherry fluorescent reporter downstream of a clock‐controlled gene promoter to explore the replicative lifespan and clock phenotypes of the *lac‐1* ^*KO*^ *bd* strain. We utilize a regression model for determining the significance of the effect *lac‐1* induction has on chronological lifespan. Finally, we examine a dosage gradient of phytoceramides to explore the longevity effect of different phytoceramide media concentrations on both *lac‐1* ^*KO*^ *bd* and WT strains under stressed conditions to test for balancing selection. In this way, we aim to establish a second line of evidence through *lac‐1* that sphingolipid biosynthesis links aging and circadian rhythms in a common stress response (Case et al., 2014).

## 2 Materials and Methods

### 2.1 Strains and media

The strains used in our chronological lifespan and replicative lifespan studies include wild‐type WT OR74A (FGSC 987), *lac‐1* ^*KO*^ *a* (NCU 02468 and FGSC 13903, Colot et al., 2006), *frq* ^*KO*^ *A* (FGSC 15070), *lac‐1* ^KO^(PB66), *lac‐1* ^*KO*^ *bd* (PB42), *lac‐1* ^*KO*^ *bd his‐3* (PB47), *bd* A (FGSC1858), and *lac‐1* ^*KO*^ *bd* (PB20i) with a functional copy of *lac‐1* ^*+*^ under the control of a quinic acid‐dependent promoter) (Table 1). Strains PB66, PB42, and PB47 were all generated from a genetic cross between *lac‐1* ^*KO*^ *a* (FGSC13903) and 87‐84‐10A (*bd his‐3*). The inducible strain PB20i was created through a spheroplast transformation of PB47 (*lac‐1* ^*KO*^ *bd his‐3*) as described in a later section.

**Table 1:** *Neurospora crassa* strains used here

Strain	Genotype	FGSC no.	Genetic background
WT OR74A	WT A	987	NA
*lac‐1* ^*KO*^ *a*	*lac‐1* ^*KO*^ *a*	13903	NA
*frq* ^*KO*^ *A*	*frq* ^*KO*^ *A*	15070	NA
87‐84‐10A	*bd his‐3 A*	NA	NA
*bd A*	*bd A*	1858	NA
PB66	*lac‐1* ^KO^	NA	*lac‐1* ^*KO*^ *a* × 87‐84‐10A
PB42	*lac‐1* ^*KO*^ *bd*	NA	*lac‐1* ^*KO*^ *a* × 87‐84‐10A
PB47	*lac‐1* ^*KO*^ *bd his‐3*	NA	*lac‐1* ^*KO*^ *a* × 87‐84‐10A
PB20i	*lac‐1* ^*KO*^ *bd (qa‐2:lac‐1* ^*+*^ *)*	NA	PB47 (transformed) × 87‐84‐10A
MFNC9	*ccg‐2p:mCherry*	10626	*NA*
PB40	*lac‐1* ^*KO*^ *ccg‐2p:mCherry*	NA	MFNC9 × *lac‐1* ^*KO*^ *a*
*frq*51	*frq* ^*KO*^ *ccg‐2p:mCherry*	NA	MFNC9 × *frq* ^*KO*^ *A*

Fluorescent strains used in this study include MFNC9 (FGSC 10626, Castro‐Longoria, Ferry, Bartnicki‐Garcia, Hasty, & Brody, 2010), *frq51,* and *lac‐1* ^*KO*^ *, MFNC9* (PB40). Strain PB40 was created from a cross between MFNC9 and *lac‐1* ^*KO*^ *a*. The *frq* ^*KO*^ *, MFNC9* (*frq51*) strain was created by a cross between MFNC9 and knockout of *frequency (frq), frq* ^*KO*^ *A*.

Media used for these experiments include 1.5% glucose media, 0.001 M quinic acid media, corn meal agar media for performing crosses, minimal carbon media for fluorescence experiments (3% sorbose, 1 M sorbitol, 0.0125% glucose, 0.0125% fructose, 1× Vogels), and sorbose + fructose + glucose (SFG) media for plating spores from crosses (Davis & de Serres, 1970).

### 2.2 Replicative lifespan: race tube experiments

All race tube media were prepared as Vogel's + 1.5% agar + 0.5% arginine using either 0.001 M QA or 1.5% glucose as the carbon source. The carbon source QA was chosen to mimic better natural conditions. Initial race tubes were started by adding 20 μl of ~10^7^ cells/ml conidial solution and filtered through cotton. The growth fronts in the tubes were marked at the same time every day under red light. Once the samples had grown to the end of the tube, their pictures were taken and digitized. In addition, the daily growth fronts and any banding activity were measured relative to the point of inoculation and entered into a FORTRAN‐IV program to calculate growth rate, period, and phase shift data (Dharmananda, 1980).

### 2.3 The use of fluorescent recorders (mCherry) to assay the effect of *lac‐1* on the clock

Fluorescent mCherry strains (*MFNC9*;* lac‐1* ^*KO*^ *, MFNC9* (PB40); *frq* ^*KO*^ *, MFNC9* (*frq51*)*)* were grown on 1.5% glucose media for 72 hr at 25°C in the dark and were allowed to conidiate on the benchtop for an additional 96 hrs. Strains were then transferred to minimal media (1.5% sorbose + 0.001 M quinic acid in 1× Vogels media, filter‐sterilized) and filtered through cotton to select conidia. Concentration was adjusted to 5–6 × 10^6^ cells/ml when possible. 200 μl of cellular suspension was added to each well in the 96‐well deep‐well plate (Marsh Biomedical Products, Inc., Rochester, NY, USA), which was then allowed to synchronize under light at room temperature (~25°C) for 26 hr. The loaded, light‐synchronized cells inserted into the fluorometer (DTX 800/88 Beckman Coulter, Inc., Fullerton, CA 92834, USA), and an experiment was run in the dark for 48 hr with measurements being taken every 30 min using 535‐nm excitation and 625‐nm emission filter using the fluorescence plate reader. All fluorescent strains were analyzed in triplicate.

Data analysis for the fluorometry experiments was performed by averaging fluorescent intensity across each triplicate set for each time point. These averages were then graphed to represent the entire 48‐hour profile for each strain. In all cases, a detrending was required to remove the effects of conidial growth from the fluorometry profile in order to make possible a spectral analysis (Bloomfield, 1976). For example, a periodogram presumes the underlying time series is stationary. There are a variety of standard ways for detrending circadian rhythm data (e.g., Izumo, Sato, Straume, & Johnson, 2006), *but since the growth is nearly perfectly linear (see* Section 3 *), the results are not going to differ from the simplest approach of subtracting a trend line*. We have calculated the effects of detrending on the periodogram (Deng et al., 2016). This detrended data set was then subjected to spectral decomposition and a Fisher periodicity test for departures from white noise (Bloomfield, 1976).

### 2.4 Cellometer experiments for measuring conidial chronological lifespan

Cellometer experiments using the Cellometer Auto2000 (Nexcelom Lawrence, MA, USA 01843) were performed using the protocol and settings for counting macroconidia only as described in the previous publication for counting macroconidia only (Case et al., 2014). Inducible strains were either grown on quinic acid agar media or on 1.5% glucose agar media for two days (30°C in the dark) and then allowed to conidiate overnight under a light at room temperature (~25°C). Conidial suspensions were created by washing cultures with sterile ddH_2_O and were equilibrated to ~3.0 × 10^6^ cells/ml using the cellometer. The suspensions were then kept in the dark for the length of the experiment.

Statistical analysis of the cellometer experiments was conducted using linear models applied to log viabilities described in the previous publication (Case et al., 2014). A separate model was utilized to analyze the effect of *lac‐1* induction using the quinic acid‐dependent promoter. The model for viability as a function of the media and strain is a minor modification of a linear model described previously (Case et al., 2014) to accommodate a slightly different experimental design. The framework of the general linear model has been extensively used to analyze selection components (Endler, 1986). In 1990, we created a more flexible and general framework for selection component analysis known as the generalized linear model for selection component experiments (Williams, Anderson, & Arnold, 1990), which included such classic analyses as those of Christiansen and Frydenberg (1976) using mother–offspring combinations and the general linear model (Endler, 1986). The advantage of this generalized linear model is being able to test a sequence of nested hypotheses about selection (Christiansen & Frydenberg, 1976; see Tables 3 and 4), solutions to overdispersion in viability counts, and resistant fitting methods (Williams et al., 1990). Here, we use a special case of the generalized linear model. The survivorship counts of cells are large (~10^3^–10^4^) so that the viability estimates are likely to be normal by the Central Limit Theorem. As a result, a simple regression is likely to approximate a life‐table analysis, and in our past experience it has worked (Case et al., 2014). For strain *i* at time *X*_*ijk*_ for day *j* on media *k*, the model is for the log viability (*Y*_*ijk*_):

(1) $$Y_{ijk}=\beta_{ik}X_{ijk}+\varepsilon_{ijk}$$

where ɛ_*ijk*_ is the measurement error. The measurement errors are assumed to be independently and identically distributed with a normal distribution having mean of 0 and variance σ^2^. The regression coefficients β_*ik*_ capture the linear relationship of log viability to time *X*_*ijk*_ for the *i*th strain in media *k*. The fitting of this model and the analysis of variance (ANOVA) used to test nested hypotheses about selection are described previously (Case et al., 2014).

The model for the experiment without a media variable is also a linear model of the same form, removing the media factor, and letting ***i** = *1, 2, or 3 to index strains compared.

### 2.5 Plasmid construction

Plasmids were constructed using pDE3dBHqa‐2 as a template (Cheng, Yang, & Liu, 2001) in *Escherichia coli* DH10α. The plasmid pDE3dBHqa‐2 contains a carbenicillin resistance cassette, a functional copy of the *histidine‐3* (*his‐3* ^*+*^) gene, and a quinic acid‐inducible promoter. The gene *lac‐1* ^*+*^ was amplified from genomic OR74A *N. crassa* DNA using primers that would incorporate an *EcoRI* restriction site at the 5′ end of the amplicon and a *ClaI* restriction site at the 3′ end of the amplicon for each gene target. We treated the plasmid and the amplicons with a double digest of *EcoRI* and *ClaI* restriction enzymes (FastDigest, Thermo Fisher Scientific, Inc. Waltham, MA, USA 02451). Following ligation of the amplicons with the linearized plasmids using T4 DNA Ligase (New England Biolabs, Inc. Ipswich, MA USA), we transformed chemically competent *E. coli* cells using the newly constructed plasmids using standard protocol. Transformed cells were plated on carbenicillin‐containing LB agar and incubated overnight at 37°C. Resulting colonies were then grown up for an additional 24 hours in carbenicillin‐containing LB liquid media. Plasmids were extracted using the High‐Pure Plasmid Isolation Kit (Roche, Inc. Indianapolis, IN, USA) and were confirmed to contain *lac‐1* by sequencing the region around the insertion. This yielded the plasmid: pDE3dBHqa‐2:*lac‐1* ^+^.

### 2.6 Creation of inducible strains

The new plasmid pDE3dBHqa‐2:*lac‐1* ^+^ was transformed into PB47 (*lac‐1* ^*KO*^ *bd his‐3*) using a spheroplasting method (Case, Schweizer, Kushner, & Giles, 1979). The resulting transformant was made homokaryotic by crossing to 87‐84‐10A (*bd his‐3*). PB20i was selected among the progeny of this cross.

### 2.7 qRT‐PCR

To analyze the effects of quinic acid induction on the quinic acid‐dependent promoter in the inducible strain PB20i, we grew two liquid cultures of PB20i and two liquid cultures of WT OR74A in 1.5% glucose growth media for 48 hours in the dark (25°C, 200 rpm). The fungal tissue was isolated from the liquid media using vacuum filtration followed by rinsing with sterile ddH_2_O. PB20i and the WT strain were then either transferred to quinic acid liquid media or returned to the 25°C shaker to grow for another 4 hr at 200 rpm. After 4 hr, the tissue was isolated from growth media using vacuum filtration and ddH_2_O rinsing, dried, and then frozen in a −80°C freezer. The tissue was then ground in liquid nitrogen, and mRNA was extracted using the Spectrum Plant Total RNA kit 50 (Sigma Aldrich, Inc. St. Louis, MO, USA). Synthesis of cDNA from the extracted mRNA was performed using a SuperScript III 1st Strand cDNA Synthesis Kit (Invitrogen, Inc. Grand Island, NY USA 18080‐051).

Reverse transcriptase quantitative PCR (RT‐qPCR) was then performed on the cDNA samples in triplicate using the ABI‐Prism 7500 with Brilliant III Ultra‐Fast SYBR Green qPCR Master Mix (Agilent Technologies, Santa Clara, CA, 95051). We used primers designed to detect *lac‐1* ^*+*^, as well as ubiquitin primers as an endogenous control (primers are in Brunson, 2015; Table 4). The relative quantity (RQ) of *lac‐1* ^*+*^ mRNA was measured relative to the endogenous control and the WT sample grown in quinic acid media for 4 hours. We measure the transcription of ubiquitin as an endogenous control to determine the relative quantification of transcripts expressed by the cell (Al‐Omari et al., 2015).

### 2.8 Phytoceramide dosage‐dependent stress‐response assay

We used a protocol we have modified from a previous study on ceramide stress signaling in *N. crassa* (Plesofsky et al., 2008). We have modified the protocol to test conidial response to an increasing gradient of phytoceramide in WT, *lag‐1* ^KO^ *bd* (MC31), and *lac‐1* ^*KO*^ *bd* (PB42) strains of *N. crassa* grown in liquid culture.

Conidia were grown on solid 1.5% glucose agar media for 48 hr in the dark at 25°C. Cells were then shifted into the light for 24 hr to ensure conidiation. Conidia were then transferred to liquid Vogel's medium with 0.05% glucose and were allowed to grow for 2 hr at 30°C (shaken at 200 rpm). Following the 2‐hr growth phase, conidial concentration was adjusted to ~1.0 × 10^6^ cells/ml and 2‐DG was added to 0.015% from a stock solution (200 mg/ml). Concentration following the 2‐hr growth period was measured using the Cellometer Auto2000 (Nexcelom). From these, 2–3 ml aliquots of conidia from each strain were taken for each phytoceramide treatment. Phytoceramide was added to the aliquots from a 20 mmol/L stock solution in ethanol, resulting in aliquots containing 0, 1, 5, 10, 20, 30, and 50 μmol/L concentrations of phytoceramide. Cells in liquid media were then transferred to a 45°C shaker (200 rpm) and grown for 44 hr. For both strains, we included an “unstressed” control grown for 44 hr at 25°C in liquid Vogel's media containing 0.05% glucose, 0.05% fructose, and 1% sorbose.

After 44 hr, all cells were stained with propidium iodide and counted using the Cellometer Auto2000 protocol described in previous publication (Case et al., 2014).

## 3 Results

### 3.1 Phenotypes of *lac‐1* ^*KO*^ *bd*

**Figure 1:**
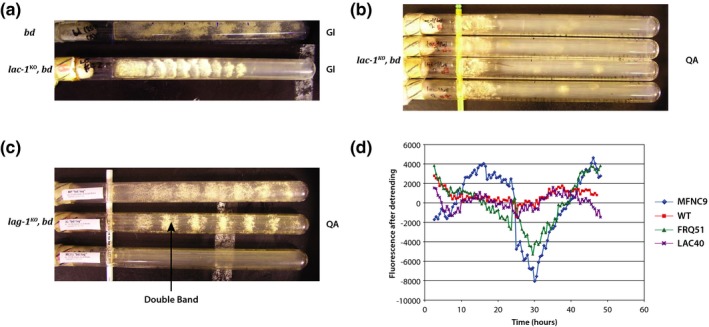
The double mutant *lac‐1* ^*KO*^ *bd* affects replicative lifespan and clock function as assayed through a *ccg‐2* promoter with a downstream fluorescent recorder gene. (a) Initial banding phenotyping of *lac‐1* ^*KO*^ *bd*, grown on glucose with a *bd* (FGSC 1858) control showing banding and growth to end of race tube. (b) Subsequent growth rate experiment of *lac‐1* ^*KO*^ *bd*, grown on quinic acid. (c) Various phenotypes of *lag‐1* ^*KO*^ *bd*, grown on quinic acid. One of the double bands is marked with an arrow. (d) loss of clock function of *lac‐1* ^*KO*^ as assayed through a fluorescent recorder downstream of *ccg‐2* promoter. Periods (±2 *SE*) of MFNC9, WT, FRQ51, LAC40, and LAC36 were estimated by fitting a sinusoid *A* cos (ω*t* + ϕ) and found to be 32 ± 2, 41 ± 8 hr, 41 ± 2, 41 ± 2, 20 ± 2, and 20 ± 2. The period of replicate 2 of MFNC9 was 28 ± 2 hr. Race tubes used glucose (GL) or quinic acid (QA) as described in Section 2

The double mutant *lac‐1* ^KO^ *bd* exhibits very slow growth on race tubes, regardless of whether quinic acid or glucose media was used. Average growth rates of 4.29 and 5.202 mm/day were observed for strains grown on glucose and quinic acid, respectively. Interestingly, the *lac‐1* ^KO^ *bd* strain exhibited signs of short replicative lifespan, such as decreased growth rate, by the end of the first race tube cycle in both the initial banding phenotyping experiment and the subsequent growth rate experiment (Figure 1a,b). In contrast, the control *bd* strain (Figure 1a) has no difficulty reaching the end of the tube in 7 days (with the time between bands being about 22 hr).

We also used race tubes to get an initial phenotype of clock function from the *lac‐1* ^KO^ *bd* strain. Although the initial banding phenotyping experiment suggests that the double mutant does have some sort of regular clock oscillations (Figure 1a), subsequent experiments revealed a very irregular banding pattern with periods ranging from 67.6 to 90.9 hr with an average of 82 hr on a different carbon source (Figure 1b). This may represent a highly senescent clock phenotype, in which replicative lifespan is no more than one race tube transfer. Other examples include strains with Kalilo elements in *N. crassa* (Griffiths, 1992) and strains of *Podospora anserina* (Osiewacz, 2002).

We have observed two altered banding phenotypes associated with *lag‐1* ^*KO*^ *bd* double mutants, suggesting disrupted clock function. In addition to the previously described “no banding” phenotype observed in the MC31 strain of *lag‐1* ^*KO*^ *bd*, we have since found a “double banding” phenotype that we have recently confirmed in two separate strains of *lag‐1* ^*KO*^ *bd* (Case et al., 2014) (Figure 1c).

We also examined by fluorometry the expression of *clock‐controlled gene‐2* (*ccg‐2*) in a *lac‐1* ^*KO*^ background over 48 hr (Figure 1d) with both positive (MFNC9) and negative controls (WT, *frequency* (*frq)* deletion with *mCherry* recorder under the control of a *ccg‐2* promoter (*frq51*)) all on the same plate. The gene *ccg‐2* was selected because it is one of the best characterized outputs of the clock (Bell‐Pedersen, Dunlap, & Loros, 1992) and has high expression (Castro‐Longoria et al., 2010; Dong et al., 2008). This *ccg‐2* promoter region has been shown to have two upstream promoter elements, one region associated with clock function and another region associated with developmental regulation (Bell‐Pedersen, Dunlap, & Loros, 1996). The reference strain (MFNC9) was engineered to lack much of the promoter element associated with developmental regulation (Castro‐Longoria et al., 2010). We verified this by sequencing the ends of the *ccg‐2* promoter region from both ends. As a consequence, MFNC9 is engineered only to give a readout from the clock.

For WT, the mean fluorescence (over three replicates, see Section 2) never exceeded 200,000 fluorescence counts, while the *lac‐1* (PB40) mutant's mean fluorescence never dropped below 250,000. The series in Figure 1d were detrended with an *R* ^2^ of 0.58 for MFNC9 and *R* ^2^ > 0.95 for remaining strains (See Section 2). The positive control (MFNC9) shows pronounced periodicity of at least 21 hr following spectral decomposition, but the remaining mutants showed no such periodicity. (The positive control was replicated in triplicate on the luminometer, and fluorescent counts displayed a strong peak in the periodogram at 22.5 hr.) For example, if one were to fit a sinusoid, *A* cos (ω*t* + ϕ), where *A* is amplitude, ω is frequency, and ϕ is phase, as a function of time *t*, to the fluorescent time series on WT or FRQ51 as in Correa et al. (2003), one would obtain an extremely long period estimate of 41 hr with a standard error of 4 and 1, respectively. Such a long period is indistinguishable from a trend and hence indicative of loss of periodicity. For example, the *lac‐1 (PB40)* mutant behaved like a *frq* knockout with the fluorescent *ccg‐2* recorder or the WT without any recorder. The amplitude of the fluctuations for *lac‐1* is similar in magnitude to that of WT (Figure 1d). So, even if *lac‐1* were periodic, it would do so with much reduced amplitude relative to the reference strain (MFNC9) (Figure 1d). None of the mutants or controls used in this experiment behaved as white noise (see Section 2). Since *lag‐1* and *lac‐1* are homologs encoding ceramide synthases and exhibit interesting clock and senescent phenotypes in race tube experiments, we were interested to discover if *lac‐1* ^KO^ *bd* also had an interesting phenotype with regard to chronological senescence.

### 3.2 Chronological lifespan of *lac‐1* ^*KO*^ *bd*: single gene effect

**Figure 2:**
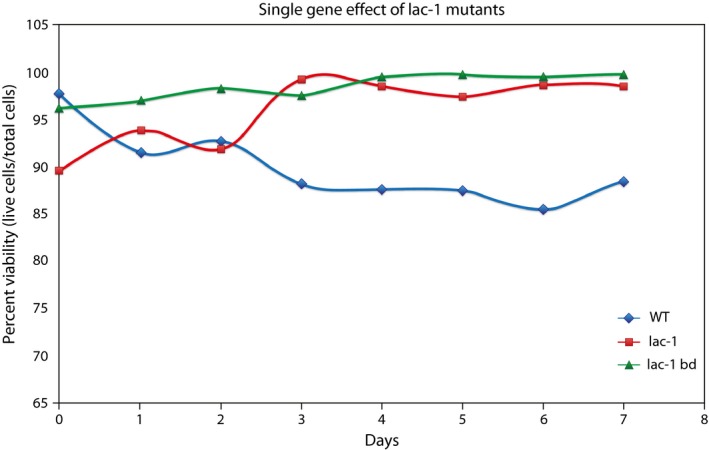
The double mutant *lac‐1* ^*KO*^ *bd* substantially increases chronological lifespan relative to wild type (WT). Viabilities of WT, *lac‐1* ^KO^, and *lac‐1* ^KO^ *bd* strains are presented as a function of time, yielding chronological lifespan estimates (Table 2). Viability is measured as a proportion of living cells to total cells, with dead cells counted using propidium iodide fluorescence measured on the Cellometer Auto2000 (Nexcelom)

After examining the replicative lifespan and clock phenotypes of *lac‐1* ^KO^ *bd*, we wanted to see how the mutations affected the viability of individual conidia. The viability of the strain was assayed with the Cellometer Auto2000 (Nexcelom) (see Section 2). We compared the viabilities of the *lac‐1* ^KO^ *bd* strain (PB42) to WT (OR74A) and the single mutant *lac‐1* ^KO^ (PB66) over the course of a 7‐day experiment with viability readings taken on the cellometer once each day at the same time of day (Figure 2).

Statistical regression analysis of the viability curves in Figure 2 revealed the average and median lifespans reported in Table 2. The expected median lifespan for WT *N. crassa* was 26.87 days, similar to a previously reported observation of 24 days (Munkres & Furtek, 1984a). However, the expected median lifespan for the *lac‐1* ^KO^ single mutant was much higher at 161.2 days, and the expected median lifespan for the double mutant was even longer at 462.1 days. We confirmed that cells in water at room temperature were still viable after 60 days. This indicates an increase in viability and in chronological lifespan, which stands in contrast with the short replicative lifespan observed in the race tubes. An ANOVA for testing viability differences between these three strains is summarized in Table 3.

**Table 2:** Estimates of mortality rates per day (log‐scale for viability), expected life span, and expected median life span for wild‐type WT (OR74A), *lac‐1* ^KO^ (PB66), and *lac‐1* ^*KO*^ *bd* (PB42) strains over 7 days

Strain	WT	*lac‐1* ^KO^	*lac‐1* ^KO^, *bd*
Death rate (*b*)	−0.0258	−0.0043	−0.0015
Standard error (*SE*)	0.0035	0.0035	0.0035
Expected average lifespan (−1/b)	38.75969	232.5581	666.6667
Expected median lifespan (−ln 2)/*b*	26.86617	161.197	462.0981

**Table 3:** There are significant differences in viability between WT (OR74A), *lac‐1* ^KO^ (PB66), and *lac‐1* ^*KO*^ *bd* (PB42) over 7 days

Description	Degrees of freedom (*df*)	Sums of squares (SS)	Estimated mean square (*EMS*)	*F*	Significance probability (*p*)
Between strains versus one viability	2	0.0493	0.0247	13.61	<.005
Viability	1	0.0466	0.0466	25.67	<.001
Error	20	0.0363	0.0018		
Corrected total	23	0.1322			*R* ^2^ = 0.73

Analysis of variance **(**ANOVA) of the log viability regression model for the three strains in Table 2, described in Section 2.

### 3.3 Induction experiment

**Figure 3:**
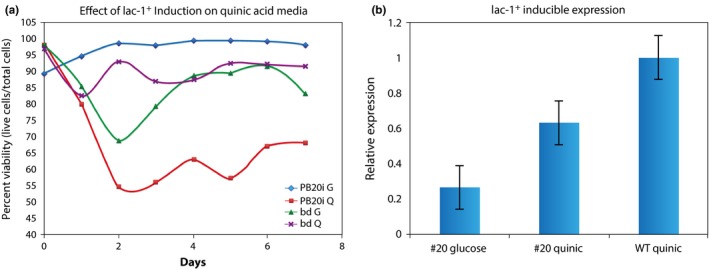
The gene *lac‐1* has a single gene effect on chronological lifespan. (a) Chronological lifespan of *bd* and the inducible *lac‐1* ^*+*^ strain (PB20) on both inducing (quinic acid (Q)) and noninducing (glucose (G)) media. Viability is measured as a proportion of living cells to total cells, with dead cells counted using propidium iodide fluorescence measured on the Cellometer Auto2000 (Nexcelom). (b) Expression of *lac‐1* ^*+*^ mRNA transcript from lac‐1^+^ (PB20) grown on glucose and PB20 grown on quinic acid, relative to *lac‐1* ^*+*^ mRNA transcript from WT OR74A grown on quinic acid. The error bars are ±2 *SE*s computed from the cDNA samples prepared in triplicate (see Section 2).

To verify the phenotypic effect of *lac‐1* knockout on chronological lifespan, we cloned the *lac‐1* ^*+*^ gene from WT OR74A genomic DNA and placed it in under the control of a quinic acid‐dependent promoter located on the plasmid pDE3dBHqa‐2 (Cheng et al., 2001). We then transformed this plasmid into a *lac‐1* ^*KO*^ *bd his‐3* strain and crossed with a *bd his‐3* strain to make the transformant homokaryotic. We tested the inducible promoters in the progeny by performing qRT‐PCR on WT strains and construct‐containing transformants following growth on either glucose media or quinic acid media (Figure 3). Expression of *lac‐1* ^*+*^ transcript was two orders of magnitude higher in the WT strain grown on glucose than it was on quinic acid, which was not surprising considering that quinic acid is a limited carbon source and a starvation response may repress the expression of certain genes (data not shown). However, we were able to detect an induction effect in the progeny strain PB20i, noting that the level of transcription as driven by the quinic acid promoter is not quite as high as the transcription being driven by the natural promoter of *lac‐1* as seen in the WT strain, yet still higher than the noninduced knockout as grown on glucose.

We then performed a viability experiment using the Cellometer Auto2000 (Nexcelom) on the *bd* strain (FGSC 1858A) and the inducible progeny PB20i, both strains grown on glucose and quinic acid for a total of four treatments (Figure 3). Selecting the *bd* strain as the control was made because it is in the double mutant *lac‐1, bd* that there is the most pronounced viability effect. To analyze this data, we used a modified version of the linear model described in the Section 2 to account for media effect, construct effect, and the effect of inducing transcription at the quinic acid‐dependent promoter. The analysis is summarized in Table 4 **.**

**Table 4:** There is a significant difference in viability between the induced and noninduced strain pDE3dBHqa‐2:*lac‐1* ^+^ in *lac‐1* ^*KO*^ *bd his‐3* (PB20i) accompanied by a media effect

Description	Degrees of freedom (*df*)	Sums of squares (SS)	Estimated mean squares (*EMS*)	*F*	Significance probability (*p*)
Difference between induced and noninduced with media effect	1	0.5615	0.5615	25.18	<.0001
Media effect and construct effect	1	0.0085	0.0085	0.38	.54
Media effect only	1	0.0677	0.0677	3.04	.09
Viability effect only	1	0.7478	0.7478	33.53	<.0001
Error	27	0.6021	0.0223		
Corrected total	31	1.9876			*R* ^2^ = 0.70

ANOVA of the log viability regression models used to analyze the effects of inducing *lac‐1* ^*+*^ transcription using the quinic acid‐dependent promoter (see Section 2).

We found that induction of *lac‐1* ^*+*^ had a highly significant effect on viability (*p* < .0001), while the introduction of the construct from the earlier transformation did not have a significant effect (*P* = .54). We would expect a significant construct effect if the plasmid had integrated into an important gene or region of the genome, but this does not seem to be the case for the transformation of pDE3dBHqa‐2:*lac‐1* ^+^ into *lac‐1* ^*KO*^ *bd his‐3*. The media effect alone was just barely insignificant (*p* = .09), and in a similar experiment on an inducible *lag‐1* ^+^ strain the media effect was not significant (unpublished results). It is worth noting that the experiments summarized in both Tables 3 and 4 have *R* ^2^ values comparable to chronological lifespan analyses on Cellometer data from our previous publication (Case et al., 2014).

Our estimated death rates revealed a strong decrease in viability after the induction of *lac‐1* ^*+*^ using the quinic acid‐dependent promoter (Table 5). The inducible strain PB20i had a very similar viability to the *lac‐1* ^*KO*^ *bd* strain previously analyzed (Tables 2 and 5), confirming the viability effect of the double mutant, but induction of *lac‐1* ^*+*^ shortened the estimated median chronological lifespan to just 7.5 days. While there may be other explanations for this sharp decrease in viability, such as interference between the induced *lac‐1* ^*+*^ transcript and other transcriptional units, there is interesting evidence that the expression of ceramide synthases, such as *lag‐1* or *lac‐1*, and the level of phytoceramide production may have been optimized by natural selection to a specific level over time, and any other level of ceramide production may be deleterious (Plesofsky et al., 2008) (Jiang et al., 2004).

**Table 5:** Summary of observed effects on viability for the *lac‐1* ^*+*^ inducible progeny PB20i and the strain *bd* 1848A, grown on both glucose and quinic acid

Condition	Construct on glucose	Construct on QA	*bd* on glucose	*bd* on QA
Death rate (*b*)	−0.0028	−0.0923	−0.0311	−0.0200
Standard error (SE)	0.0124	0.0124	0.0124	0.0124
Expected average life span (−1/*b*)	357.1429	10.83424	32.15434	50
Expected median life span (−ln 2)/*b*	247.5526	7.50972	22.28769	34.65736

Death rates were calculated from our regression analyses as summarized in Table 4.

### 3.4 Ceramide experiment

**Figure 4:**
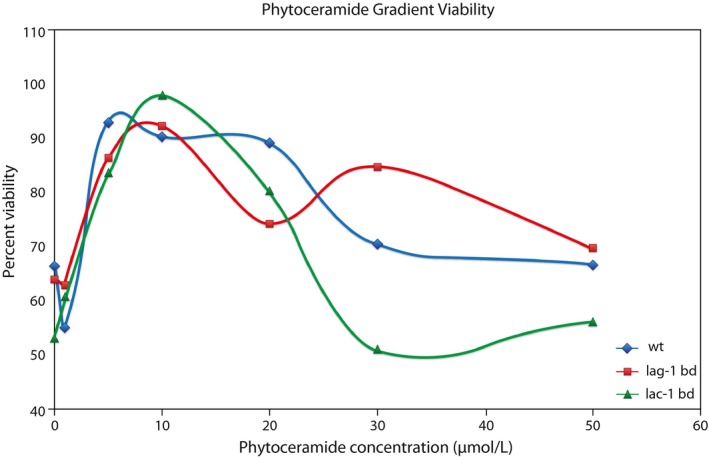
The level of phytoceramide is under optimizing selection. Effect of a phytoceramide dosage gradient on WT, *lag‐1* ^*KO*^ *bd*, and *lac‐1* ^*KO*^ *bd* strains. Viability is measured as a proportion of living cells to total cells, with dead cells counted using propidium iodide fluorescence measured on the Cellometer Auto2000 (Nexcelom). The lac‐1,ras‐1 double mutant of Neurospora crassa replicatively senesces as it grows from one end of the “race tube” and stalls about ~2/3 of the way down the “race tube.”

Our findings from the *lac‐1* ^+^ inducible experiment prompted us to explore the effects of phytoceramide dosage on WT, the *lac‐1* ^*KO*^ *bd* double mutant PB42, and a *lag‐1* ^*KO*^ *bd* double mutant (MC31) described in our previous publication (Case et al., 2014). Previous studies have suggested that phytoceramide is expressed at an optimum level within the conidia of *N. crassa* (Plesofsky et al., 2008). We predict that the optimum level of phytoceramide production shifts based on the activity of phytoceramide‐producing genes. In the event that genes responsible for the synthesis of phytoceramide are knocked out, the optimum conidial viability should shift to a higher dosage of phytoceramides, since phytoceramide is being produced at a lower level, or not at all, within the ceramide synthase knockout mutants. To test this hypothesis, we designed an experiment akin to a previous study on the effects of phytoceramide dosage and stress response, except here we have incorporated the use of the Cellometer Auto2000 for viability measurements instead of using standard plating protocol (Plesofsky et al., 2008). Results of this experiment are summarized in Figure 4.

While all three genotypes have absolute maximum viabilities at ~10 μmol/L phytoceramide, the *lag‐1* ^KO^ *bd* double mutant approaches a second maximum at a phytoceramide concentration of ~30 μmol/L. This dosage effect represents interesting additional evidence for balancing selection acting on phytoceramide concentration, since a stabilizing effect can be witnessed even in the absence of an important ceramide synthase and is distinguishable from the stabilizing effect observed in a WT strain treated with the same dosage gradient of phytoceramide. The dosage–response curve *lac‐1* ^KO^ *bd* double mutant closely mirrors the curve for the WT strain, so we cannot conclude that the absence or lack of phytoceramide production is creating the longevity effect observed in *lac‐1* ^KO^ *bd* mutant. The dosage response of *lac‐1* ^*KO*^ *bd*, for example, is:

$$\begin{array}{cc} \text{Log viability}= & 53.52\left(\pm 1.5807\right)+8.1198\left(\pm .4298\right)X-.4463\left(\pm .0236\right)X^{2}\\ & +.0057\left(\pm .0003\right)X^{3}\left(\text{with}\;R^{2}>.99\right), \end{array}$$

where *X* is the phytoceramide concentration. The fit is nearly perfect, and all of the regression coefficients (e.g., −0.4463) on phytoceramide concentration and its powers are highly significant. The intercepts of the other two dosage–response curves are similar in shape, but the intercepts are quite different with WT and *lag‐1* ^*KO*^ *,bd* being 4.1030 ± 0.0985 and 64.57 ± 7.86, respectively. So, clearly the mutant curves are significantly different from WT, although they mirror each other.

## 4 Discussion

Case et al. (2014) provided one line of evidence that aging and circadian rhythms as stress responses are linked through *lag‐1*; its homolog *LAG1* in *S. cerevisiae* was the first longevity gene to be characterized in yeast (D'mello et al., 1994). The homologs to *LAG1* in *Caenorhabditis elegans*,* Homo sapiens, and N. crassa* were later shown to complement *LAG1* (Case et al., 2014; Jiang, Kirchman, Zagulski, Hunt, & Jazwinski, 1998), and so *LAG1* is likely to have a highly conserved role in aging in eukaryotes. The double mutant, *LAG1,LAC1*, appears not to produce any sphingolipids, such as ceramides (Schorling, Valle, Barz, Riezman, & Oesterhelf, 2001).

Here, *both lag‐1* ^KO^ *bd* and *lac‐1* ^KO^ *bd* have substantial effects on chronological lifespan and clock phenotype in *N. crassa*, consistent with the hypothesis that sphingolipid metabolism links aging and circadian rhythms in a common stress response. In both genotypes, we find that chronological lifespan is significantly increased versus the wild type as measured using automated cell counting protocol (Berkes et al., 2012). Unexpectedly, the *lac‐1* ^KO^ *bd* double mutant seems to increase chronological lifespan (Table 2 **),** while at the same time decreasing replicative lifespan (Figure 1). The *lag‐1* ^KO^ *bd* double mutant has similar phenotypes with regards to chronological and replicative lifespan (Case et al., 2014). This is not without precedent. The *RAS1* paralog, *RAS2*, in *S. cerevisiae* also simultaneously increases chronological lifespan and decreases replicative lifespan (Fabrizio et al., 2003; Sun, Kale, Childress, Pinswasdi, & Jazwinski, 1994). Chronological aging and replicative aging, however, are not the same traits. In fact, in *S. cerevisiae* they appear to be under the control of distinct suites of genes (Stumpferl et al., 2012) with a handful of genes affecting both (Fabrizio et al., 2003; Sun et al., 1994).

We also conclude that the concentration of phytoceramide is responsible, at least in part, for the altered longevity phenotype in the *lag‐1* ^KO^ *bd* strain. In the WT control strain, we observe a different optimal concentration than what has been reported previously (Plesofsky et al., 2008). This is not unexpected, since phytoceramide is being administered in two different ways in the two experiments: In our experiment we expose conidia to phytoceramide in liquid culture as opposed to on agar plates. In addition, our protocol for estimating chronological lifespan using automated cell counting is specific to viability of macroconidia, whereas a plating method would not employ such specificity. However, regardless of the method used, we are still able to observe the optimization of phytoceramide in *N. crassa* conidia, confirming previous observations as well as allowing us to recognize subtle shifts in viability profiles as a result of genetic knockouts (Figure 4). This demonstrates that our automated cell counter‐based estimates of chronological lifespan could be reasonably adapted to a wide variety of assays and experiments, especially considering we have already demonstrated its accuracy in viability estimates (Case et al., 2014).

While we have not identified the specific biochemical cause of increased chronological longevity among *lac‐1* ^KO^ *bd* mutants, we have shown that the phytoceramide‐producing property of its homolog *lag‐1* is an important contributor to the longevity effect seen in *lag‐1* ^KO^ *bd*. Since *lag‐1* ^KO^ *bd* also has an altered clock phenotype, phytoceramide might play a role in regulating clock function in *N. crassa* (Case et al., 2014). A separate ceramide synthesized by *lac‐1* may account for the increased chronological lifespan of *lac‐1* ^KO^ *bd* strains and could be identified using similar dosage gradients used in the phytoceramide experiments above (Figure 4 **).**

Pinpointing the role phytoceramide might play in clock regulation will require several important considerations. For example, is cell‐to‐cell communication required for proper clock functioning and circadian synchronization? If this is the case, then phytoceramide or other signaling molecules may be important for clock function, and situations in which individual conidia have no other cells to communicate with may require media supplementation with the proper signaling molecule. This is a good rationale for developing microfluidics devices for measuring the clock and longevity (Deng et al., 2016). Using microfluidics, cells could be separated into droplets of media containing differing numbers of conidia. If cellular signaling plays a role in either longevity or the clock, then droplets containing one or two conidia should display longevity and clock phenotypes distinct from droplets containing many conidia. Furthermore, if phytoceramide were the compound responsible for synchronization of clock oscillation, a dosage scheme similar to the one employed in Figure 4 would prove useful in designing a microfluidics experiment for assaying the clock.

Microfluidics technologies would also solve some of the issues with our current cellometer‐based longevity assays (Deng et al., 2014, 2016; Lee, Lee, & Hong, 2013). In our development of a protocol for assaying cell survivability in suspensions of approximately 3.0 × 10^6^ cells/ml, we found that the number of viable cells decreased dramatically over the course of experimental day 0, but returned to a viability percentage consistent with a typical linear regression model by the 24‐hr time point was taken (as described in Section 2). Our day zero analyses have shown that conidia producing hyphae are most likely to die at the start of the experiment, contributing significantly to the decline in overall viability of the population (unpublished data). While we have not posited a mechanism for the subsequent rise in viability leading up to day 1, we imagine that this mechanism might continue to play a role throughout the course of the experiment, especially if the rise in viability is linked to the utilization of organic matter released from dead cells. Cell‐sorting microfluidics could circumvent this issue by segregating cells into droplets containing one or only a few conidia and would remove substantial noise from the data in addition to improving measurements for clock oscillations in *N. crassa* mutants. These mutants could theoretically contribute either to basic clock functioning or to cell‐to‐cell signaling important for synchronization of individual biological clocks.

## Conflict of Interest

None declared.

## References

[ece32554-bib-0001] Al‐Omari, A. , Griffith, J. , Judge, M. , Taha, T. , Arnold, J. , & Schuttler, H.‐B. (2015). Discovering regulatory network topologies using ensemble methods on GPGPUs with special reference to the biological clock of *Neurospora crassa* . IEEE Access, 3, 27–42. doi: 10.1109/ACCESS.2015.2399854

[ece32554-bib-0002] Belden, W. J. , Larrondo, L. F. , Froehlich, A. C. , Shi, M. , Chen, C. H. , Loros, J. J. , et al. (2007). The band mutation in *Neurospora crassa* is a dominant allele of *ras‐1* implicating RAS signaling in circadian output. Genes & Development, 21, 1494–1505. 17575051 10.1101/gad.1551707 PMC1891427

[ece32554-bib-0003] Bell‐Pedersen, D. , Dunlap, J. C. , & Loros, J. J. (1992). The Neurospora circadian clock‐controlled gene, ccg‐2, is allelic to eas and encodes A fungal hydrophobin required for formation of the conidial rodlet layer. Genes & Development, 6, 2382–2394. 1459460 10.1101/gad.6.12a.2382

[ece32554-bib-0004] Bell‐Pedersen, D. , Dunlap, J. C. , & Loros, J. J. (1996). Distinct cis‐acting elements mediate clock, light, and developmental regulation of the *Neurospora crassa eas* (*ccg‐2)* gene. Molecular and Cell Biology, 16, 513–521. 10.1128/mcb.16.2.513 PMC231029 8552078

[ece32554-bib-0501] Bell‐Pedersen, D. , Cassone, V. M. , Earnest, D.J. , Golden, S. S. , Hardin, P. E. , Thomas, T. L. , Zoran, M. J. (2005). Circadian rhythms from diverse organisms. Nat. Rev. Genet. 6 (7): 544‐556. 15951747 10.1038/nrg1633 PMC2735866

[ece32554-bib-0005] Berkes, C. A. , Chan, L. L.‐Y. , Wilkinson, A. , & Paradis, B. (2012). Rapid quantification of pathogenic fungi by Cellometer image‐based cytometry. J. Microbio. Methods, 91, 468–476. 10.1016/j.mimet.2012.09.008 22985717

[ece32554-bib-0006] Bitterman, K. J. , Medvedik, O. , & Sinclair, D. A. (2003). Longevity regulation in *Saccharomyces cerevisiae*: Linking metabolism, genome stability, and heterochromatin. Microbiology and Molecular Biology Reviews, 67, 376–379. 12966141 10.1128/MMBR.67.3.376-399.2003 PMC193872

[ece32554-bib-0007] Bloomfield, P. (1976). Fourier analysis of time series: An introduction. New York, NY: Wiley.

[ece32554-bib-0008] Brunson, J. K. (2015). Sphingolipid synthesis and the aging biological clock in Neurospora crassa (p. 20). University of Georgia Honors Dissertation, Athens, GA: University of Georgia.

[ece32554-bib-0009] Case, M. E. , Griffith, J. , Dong, W. , Tigner, I. L. , Gaines, K. , Jiang, J. C. , … Arnold, J. (2014). The aging biological clock in *Neurospora crassa* . Ecology and Evolution, 4(17), 3494–3507. 25535564 10.1002/ece3.1202 PMC4228622

[ece32554-bib-0010] Case, M. E. , Schweizer, M. , Kushner, S. R. , & Giles, N. H. (1979). Efficient transformation of *Neurospora crassa* by utilizing hybrid plasmid DNA. PNAS USA, 76, 5259–5263. 159454 10.1073/pnas.76.10.5259 PMC413120

[ece32554-bib-0011] Castro‐Longoria, E. , Ferry, M. , Bartnicki‐Garcia, S. N. , Hasty, J. , & Brody, S. (2010). Circadian rhythms in *Neurospora crassa*: Dynamics of the clock component frequency visualized using a fluorescent reporter. Fungal Genetics and Biology, 47, 332–341. 20051268 10.1016/j.fgb.2009.12.013 PMC2935182

[ece32554-bib-0012] Cheng, P. , Yang, Y. , & Liu, Y. (2001). Interlocked feedback loops contribute to the robustness of the Neurospora circadian clock. Proceedings of the National Academy of Sciences of the United States of America, 98, 7408–7413. 11416214 10.1073/pnas.121170298 PMC34682

[ece32554-bib-0013] Christiansen, F. B. , & Frydenberg, O. (1976). Selection component analysis of natural polymorphisms using population samples including mother‐offspring combinations. Theor. Pop. Biol., 4, 425–445. 10.1016/0040-5809(73)90019-1 4779108

[ece32554-bib-0014] Colot, H. V. , Park, G. , Turner, G. E. , Ringelberg, C. , Crew, C. M. , Litvinkova, L. , … Dunlap, J. C. (2006). “A high‐throughput gene knockout procedure for *Neurospora* reveals functions for multiple transcription factors” (vol 103, pg 10352, 2006). Proceedings of the National Academy of Sciences of the United States of America, 103, 10352–10357. 16801547 10.1073/pnas.0601456103 PMC1482798

[ece32554-bib-0015] Correa, A. , Lewis, Z. A. , Green, A. V. , March, I. J. , Gomer, R. H. , & Bell‐Pedersen, D. (2003). PNAS USA, 100, 13597–135602. 14597725 10.1073/pnas.2233734100 PMC263859

[ece32554-bib-0016] Davis, R. H. , & de Serres, F. J. (1970). Genetic and microbiological research techniques for *Neurospora crassa* . Methods in Enzymology, 17, 79–143.

[ece32554-bib-0017] Deng, Z. , Arsenault, S. , Caranica, C. , Griffith, J. , Zhu, T. , Al‐Omari, A. , … Mao, L . (2016). Synchronizing stochastic circadian oscillators in single cells of Neurospora crassa, Scientific Reports 6, 35828, DOI: 10.1038/srep35828 27786253 10.1038/srep35828 PMC5082370

[ece32554-bib-0018] Deng, Z. , Arsenault, S ., Zhu, T ., Cheng, R. , Griffith, J. , … Mao, L. 2014 Singlecell measurements on the biological clock by microfluidics Proceedings of the 18th international conference on miniaturized systems for chemistry and life sciences (MicroTAS). London: Royal Society of Chemistry, San Antonio, TX 881–883. https://www.google.com/?gws_rd=ssl#q=jonathan+arnold+uga&start=2

[ece32554-bib-0019] Dharmananda, S. (1980). Studies of the circadian clock of Neurospora crassa: light‐induced phase shifting. Santa Cruz: University of California.

[ece32554-bib-0020] D'mello, N. P. , Childress, A. M. , Franklin, D. S. , Kale, S. P. , Pinswasdi, C. , & Jazwinski, S. M. (1994). Cloning and characterization of LAG& a longevity‐assurance gene in yeast. Journal of Biological Chemistry, 269, 15451–15459. 8195187

[ece32554-bib-0021] Dong, W. , Tang, X. , Yu, Y. , Nilsen, R. , Kim, R. , Griffith, J. , Arnold, J. , & Schuttler, H. B. (2008). PLoS ONE, 3(8), e3105. 18769678 10.1371/journal.pone.0003105 PMC2518617

[ece32554-bib-0022] Endler, J. A. (1986). Natural selection in the wild. Princeton, NJ: Princeton University Press.

[ece32554-bib-0023] Fabrizio, P. , Liou, L.‐L. , Moy, V. N. , Diaspro, A. , Valentine, J. S. , Gralla, E. B. , & Longo, V. D. (2003). *SOD2* functions downstream of Sch9 to extend longevity in yeast. Genetics, 163, 35–46. 12586694 10.1093/genetics/163.1.35 PMC1462415

[ece32554-bib-0024] Griffiths, A. J. F. (1992). Fungal senescence. Ann. Rev. Genet., 26, 351–372. 1482117 10.1146/annurev.ge.26.120192.002031

[ece32554-bib-0025] Guillas, I. , Kirchman, P. A. , Chuard, R. , Pfefferli, M. , Jiang, J. C. , Jazwinski, S. M. , & Conzelmann, A. (2001). C26‐CoA‐dependent ceramide synthesis of *Saccharomyces cerevisiae* is operated by Lag1p and Lac1p. The EMBO Journal, 20(11), 2655–2665. 11387200 10.1093/emboj/20.11.2655 PMC125493

[ece32554-bib-1000] Gyongyosi, N and K. Kaldi (2014). Interconnections of reactive oxygen species homeostasis and circadian rhythm in Neurospora crassa. Antioxidants and Redox Signaling 20(18): 3007‐3023. 23964982 10.1089/ars.2013.5558

[ece32554-bib-0026] Hannun, Y. , & Obeid, L. (2011). Many ceramides. Journal of Biological Chemistry, 286(32), 27855–27862. 21693702 10.1074/jbc.R111.254359 PMC3151029

[ece32554-bib-0027] Izumo, M. , Sato, T. R. , Straume, M. , & Johnson, C. H. (2006). Quantitative analyses of circadian gene expression in mammalian cell cultures. PLoS Computational Biology, 2, e136. 17040123 10.1371/journal.pcbi.0020136 PMC1599765

[ece32554-bib-0028] Jiang, J. C. , Kirchman, P. A. , Allen, M. , & Jazwinski, S. M. (2004). Suppressor analysis points to the subtle role of the LAG1 ceramide synthase gene in determining yeast longevity. Experimental gerontology, 39(7), 999–1009. 15236759 10.1016/j.exger.2004.03.026

[ece32554-bib-0029] Jiang, J. C. , Kirchman, P. A. , Zagulski, M. , Hunt, J. , & Jazwinski, S. M. (1998). Homologs of the yeast longevity gene LAG1 in *Caenorhabditis elegans* and human. Genome Research, 8, 1259–1272. 9872981 10.1101/gr.8.12.1259

[ece32554-bib-0030] Judge, M. , Griffith, J. , & Arnold, J. (2017). Aging and the biological clock In Jazwinski M. J., Balancio V. P., & Hill S. M. (Eds.), Circadian rhythms and their impact on aging. Dordecht, Netherlands: Springer Science + Business Media.

[ece32554-bib-0031] Lakin‐Thomas, P. L. , & Brody, S. (2000). Circadian rhythms in *Neurospora crassa*: lipid deficiencies restore robust rhythmicity to null frequency and white‐collar mutants. Proceedings of the National Academy of Sciences, 97(1), 256–261. 10.1073/pnas.97.1.256 PMC26650 10618405

[ece32554-bib-0032] Lee, K. , Lee, C. H. , & Hong, C. (2013). Circadian rhythms in *Neurospora crassa* on a microfluidic device for real‐time gas perturbations In Morante J. R. & Hierold C. (Eds.), Solid‐state sensors, actuators and microsystems (TRANSDUCERS & EUROSENSORS XXVII), 2013 transducers & eurosensors XXVII: The 17th international conference on (pp. 1247–1250). Piscataway: IEEE.

[ece32554-bib-0033] Lerner, I. (1954). Genetic homeostasis. New York, NY: Wiley.

[ece32554-bib-0034] Merrill, A. H. Jr (2002). *De novo* sphingolipid biosynthesis: A necessary, but dangerous pathway. Journal of Biological Chemistry, 277, 25843–25846. 12011104 10.1074/jbc.R200009200

[ece32554-bib-0035] Munkres, K. D. , & Furtek, C. A. (1984a). Selection of conidial longevity mutants of *Neurospora crassa* . Mechanisms of Aging and Development, 25, 47–62. 10.1016/0047-6374(84)90129-5 6233461

[ece32554-bib-0036] Munkres, K. D. , & Furtek, C. A. (1984b). Assay of rate of aging of conidia of *Neurospora crassa* . Methods in Enzymology, 105, 263–270. 6233470 10.1016/s0076-6879(84)05034-5

[ece32554-bib-0037] Osiewacz, H. (2002). Mitochondrial functions and aging. Gene, 286, 65–71. 11943461 10.1016/s0378-1119(01)00804-6

[ece32554-bib-0038] Ottesen, E. A. , Young, C. R. , Gifford, S. M. , Eppley, J. M. , Marin, R. , Schuster, S. C. , … DeLong, E. F. (2014). Multispecies diel transcriptional oscillations in open ocean heterotrophic bacterial assemblages. Science, 345(6193), 207–212. 25013074 10.1126/science.1252476

[ece32554-bib-0039] Plesofsky, N. S. , Levery, S. B. , Castle, S. A. , & Brambl, R. (2008). Stress‐induced cell death is mediated by ceramide synthesis in Neurospora crassa. Eukaryotic Cell, 7(12), 2147–2159. 18952903 10.1128/EC.00147-08 PMC2593191

[ece32554-bib-0040] Rose, M. R. (1991). Evolutionary biology of aging. New York, NY: Oxford University Press.

[ece32554-bib-0041] Schluter, D. (1988). Estimating the form of natural selection on a quantitative trait. Evolution, 42(5), 849–861. 10.1111/j.1558-5646.1988.tb02507.x 28581168

[ece32554-bib-0042] Schorling, S. , Valle, B. , Barz, W. P. , Riezman, H. , & Oesterhelf, D. (2001). Lag1p and Lac1p are essential for the Acyl‐CoA–dependent ceramide synthase reaction in *Saccharomyces cerevisiae* . Molecular Biology of the Cell, 12, 3417–3427. 11694577 10.1091/mbc.12.11.3417 PMC60264

[ece32554-bib-0043] Shi, M. , Larrondo, L. F. , Loros, J. J. , & Dunlap, J. C. (2007). A developmental cycle masks output from the circadian oscillator under conditions of choline deficiency in Neurospora. PNAS USA, 104, 20102–20107. 18056807 10.1073/pnas.0706631104 PMC2148429

[ece32554-bib-0044] Slack, C. , Alic, N. , Foley, A. , Cabecinha, M. , Hoddinott, M. P. , & Partridge, L. (2015). The Ras‐Erk‐ETS‐signaling pathway is a drug target for longevity. Cell, 162, 72–83. 26119340 10.1016/j.cell.2015.06.023 PMC4518474

[ece32554-bib-0045] Stinchcombe, J. , Agrawal, A. , Hohenlohe, P. , Arnold, S. , & Blows, M. (2008). Estimating nonlinear selection gradients using quadratic regression coefficients: double or nothing? Evolution, 62(9), 2435–2440. 18616573 10.1111/j.1558-5646.2008.00449.x

[ece32554-bib-0046] Stumpferl, S. W. , Brand, S. E. , Jiang, J. C. , Korona, B. , Tiwari, A. , Dai, J. , … Jazwinski, S. M. (2012). Natural genetic variation in yeast longevity. Genome Research, 22(10), 1963–1973. 22955140 10.1101/gr.136549.111 PMC3460191

[ece32554-bib-0047] Sun, J. , Kale, S. P. , Childress, A. M. , Pinswasdi, C. , & Jazwinski, S. M. (1994). Divergent roles of *RAS1* and *RAS2* in yeast longevity. Journal of Biological Chemistry, 269, 18638–18645. 8034612

[ece32554-bib-0048] Warnecke, D. , & Heinz, E. (2003). Recently discovered functions of glucosylceramides in plants and fungi. Cellular and Molecular Life Sciences, 60, 919–941. 12827281 10.1007/s00018-003-2243-4 PMC11138565

[ece32554-bib-0049] Williams, C. J. , Anderson, W. W. , & Arnold, J. (1990). Generalized linear modeling methods for selection component experiments. Theor. Pop. Biol., 37, 389–423.

